# Reactivity of Thiol-Rich Zn Sites in Diacylglycerol-Sensing PKC C1 Domain Probed by NMR Spectroscopy

**DOI:** 10.3389/fmolb.2021.728711

**Published:** 2021-08-10

**Authors:** Taylor R. Cole, Tatyana I. Igumenova

**Affiliations:** Department of Biochemistry and Biophysics, Texas A&M University, College Station, TX, United States

**Keywords:** protein kinase C, C1 domain, zinc finger, cadmium, thiol-rich sites, cysteine reactivity, NMR spectroscopy, metal ion toxicity

## Abstract

Conserved homology 1 (C1) domains are peripheral zinc finger domains that are responsible for recruiting their host signaling proteins, including Protein Kinase C (PKC) isoenzymes, to diacylglycerol-containing lipid membranes. In this work, we investigated the reactivity of the C1 structural zinc sites, using the cysteine-rich C1B regulatory region of the PKCα isoform as a paradigm. The choice of Cd^2+^ as a probe was prompted by previous findings that xenobiotic metal ions modulate PKC activity. Using solution NMR and UV-vis spectroscopy, we found that Cd^2+^ spontaneously replaced Zn^2+^ in both structural sites of the C1B domain, with the formation of all-Cd and mixed Zn/Cd protein species. The Cd^2+^ substitution for Zn^2+^ preserved the C1B fold and function, as probed by its ability to interact with a potent tumor-promoting agent. Both Cys_3_His metal-ion sites of C1B have higher affinity to Cd^2+^ than Zn^2+^, but are thermodynamically and kinetically inequivalent with respect to the metal ion replacement, despite the identical coordination spheres. We find that even in the presence of the oxygen-rich sites presented by the neighboring peripheral membrane-binding C2 domain, the thiol-rich sites can successfully compete for the available Cd^2+^. Our results indicate that Cd^2+^ can target the entire membrane-binding regulatory region of PKCs, and that the competition between the thiol- and oxygen-rich sites will likely determine the activation pattern of PKCs.

## Introduction

Approximately ∼10% of the human proteome uses Zn^2+^ as a cofactor (Andreini et al., 2006). While Zn^2+^ is not redox active, it plays a critical role in many vital cellular processes. Functional annotation of Zn proteome predicts a wide range of biological and enzymatic activities (Bertini et al., 2010), with over 40% of the assigned sequences involved in the regulation of gene expression. One of the key signaling enzymes that require Zn^2+^ is the family of Protein Kinase C isoenzymes (PKCs). By serving as the key node in the phosphoinositide signaling pathway, PKCs regulate cell growth and differentiation (Dempsey et al., 2000; Newton, 2010). Aberrant PKC activity has been implicated in many human diseases including cancer progression (Antal et al., 2015; Rahimova et al., 2020), diabetes (Koya and King, 1998; Mishra and Dey, 2021), as well as neurological (Khan et al., 2009) and cardiovascular dysfunctions (Johnson et al., 1995; Budas et al., 2007; Churchill et al., 2008; Drosatos et al., 2011). Exposure to divalent xenobiotic metal ions, such as Pb^2+^ (Markovac and Goldstein, 1988; Tomsig and Suszkiw, 1995; Sun et al., 1999; Morales et al., 2011) and Cd^2+^ (Beyersmann et al., 1994; Morales et al., 2013b) modulates PKC activity. Specifically, Cd^2+^ can exert both activating and inhibitory effects on PKCs (Block et al., 1992; Beyersmann et al., 1994; Long, 1997) (Saijoh et al., 1988; Speizer et al., 1989). Cadmium(II) is a known carcinogen (Waalkes and Rehm, 1992; Jarup et al., 1998; Waalkes, 2003; Faroon et al., 2012) with elevated levels in the environment due to human activity. The deleterious effects of cadmium are compounded by its relatively long half-life in the human body (Faroon et al., 2012). The molecular mechanism of how Cd^2+^ modulates PKC activity remains unresolved.

**FIGURE 1:**
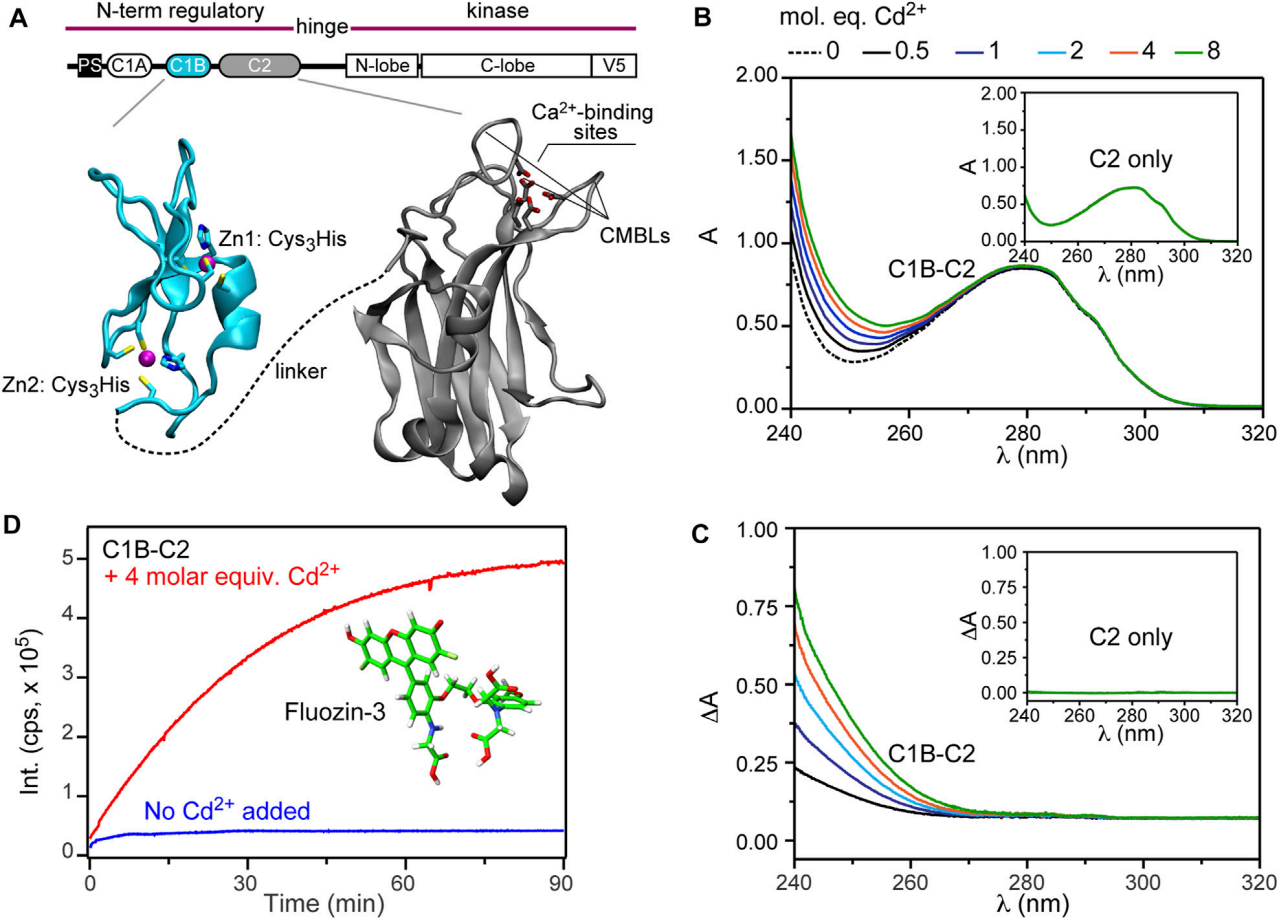
Cd^2+^ replaces Zn^2+^ in the C1B domain. **(A)** Ribbon diagrams of C1B (2ELI) and C2 (4L1L) highlighting the metal-ion ligands. CMBLs stand for Ca^2+^- and membrane-binding loop loops. **(B)** UV-vis absorption spectra for the Cd^2+^ titration of 25 μM C1B-C2. Inset: UV-vis absorption spectra for the Cd^2+^ titration of 25 μM isolated C2 domain. The spectrum of free Cd^2+^ served as the reference and was subtracted from each spectrum. **(C)** Difference UV-vis absorption spectra between C1B-C2 and C2 obtained at increasing molar equivalents of Cd^2+^. The position of the absorption shoulder is consistent with the formation of the Cd^2+^-thiolate bonds. **(D)** Cd^2+^-stimulated Zn^2+^ release from the C1B-C2 domain monitored using fluorescence intensity of FluoZin-3 (Pubchem CID 101165894) at λ = 516 nm. The no-Cd^2+^ control is shown in blue.

The regulatory domain of conventional (i.e., Ca^2+^-dependent) PKC isoforms consists of three peripheral membrane binding modules: the tandem C1A and C1B domains that penetrate the membrane in response to binding a signaling lipid, diacylglycerol, and the C2 domain that binds to anionic phospholipids in a Ca^2+^ dependent manner (Figure 1A). The membrane recruitment step, mediated by both C1 and C2, removes the autoinhibition of the enzyme and enables it to phosphorylate its targets. C1 and C2 make use of two metal-ion cofactors: Zn^2+^ and Ca^2+^, respectively. The Zn^2+^ ions, 2 per C1 domain, are coordinated by the Cys_3_His motifs each in a tetrahedral geometry (Hubbard et al., 1991; Hommel et al., 1994; Zhang et al., 1995) and are essential for the 3D fold of C1 domains. Ca^2+^ ions are required for the membrane-binding function of C2 but not for its fold (Verdaguer et al., 1999; Morales et al., 2011). Up to three Ca^2+^ ions can bind to the all-oxygen coordination site harbored by the apical loops of C2.

In this work, we applied solution NMR spectroscopy to probe Cd^2+^ interactions with the regulatory region from PKCα, with the primary objective to evaluate the reactivity of the thiol-rich Zn^2+^-coordinating sites towards Cd^2+^ substitution. For our experiments, we chose the two-domain unit from PKCα (denoted C1B-C2) that comprises C1B and the neighboring C2 connected by the native linker region (Figure 1A). C1B-C2 represents the minimum membrane-binding unit of PKCα capable of coincidence detection of four signaling molecules: diacylglycerol (C1B) and Ca^2+^/phosphatidylserine/phosphatidylinositol-4,5-bisphosphate (C2). We found that Cd^2+^ readily displaces Zn^2+^ at both structural sites in C1B, and that this process successfully competes with the Cd^2+^ interactions with the oxygen-rich C2 sites. Furthermore, despite the identical coordination spheres, the two Zn^2+^ sites show different thermodynamics and kinetics of Cd^2+^ binding. C1 and C2 domains are the basic building blocks of more than 100 proteins involved in signal transduction. Hence, the knowledge gained from this study will be applicable to other C1- and C2-containing proteins (Lemmon, 2008), leading to a more complete understanding of how xenobiotic metal ions interfere with the mechanisms of signal transduction and elicit a toxic response.

## Results

### Cd^2+^ Coordinates Thiol Groups and Ejects Zn^2+^ From C1B

The first step was to determine how Cd^2+^ interacts with the C1B-C2 domain using UV-vis absorption spectroscopy. It is well established that thiolate-Cd^2+^ charge transfer bands have characteristic wavelengths at around ∼240 nm (Busenlehner et al., 2001; Habjanič et al., 2020). The C1B domain has six cysteine residues, all of which are involved in coordinating the structural Zn^2+^ ions (Figure 1A). C2 is cysteine-free, but can bind Cd^2+^ with high affinity through the vacant oxygen-rich sites formed by the aspartate carboxyl groups and the carbonyl oxygens of W247 and M186 (Morales et al., 2013a). Thus, the presence of thiolate-Cd^2+^ charge transfer bands upon C1B-C2 treatment with Cd^2+^ can only originate from Cd^2+^ coordinating Cys residues of C1B.

Addition of increasing amounts of Cd^2+^ to C1B-C2 resulted in significant spectral changes (Figure 1B). Based on the C2-only control experiment with Cd^2+^ (inset of Figure 1B), these changes can only be attributed to the C1B-Cd^2+^ interactions. The difference UV-Vis spectra, where the protein contribution to the absorbance is subtracted out, clearly shows the build-up of a shoulder near *λ* = 270 nm (Figure 1C). The wavelength range is consistent with the position of thiolate-Cd^2+^ charge transfer bands observed in other studies (Busenlehner et al., 2001; Habjanič et al., 2020). Based on this information and previous work on the Zn^2+^-containing proteins with Cys-rich sites (Wang et al., 2005; Chakraborty et al., 2011; Malgieri et al., 2011), we conclude that Cd^2+^ forms coordination bonds with the cysteine residues of C1B, even in the presence of Cd^2+^-sequestering C2.

Two scenarios are possible: Cd^2+^ can either eject and substitute for Zn^2+^, or Cd^2+^ can peripherally coordinate cysteines without displacing Zn^2+^, forming a binuclear metal cluster similar to that observed in the GAL4 transcription factor (Pan and Coleman, 1990). To distinguish between these two scenarios, we used a highly selective Zn^2+^ fluorophore, FluoZin-3. Four molar equivalents of Cd^2+^ were added to the C1B-C2 domain in the presence of FluoZin-3, and the time-dependent fluorescence intensity was monitored at 516 nm. We observed a steady increase in the fluorescence intensity, indicating that Zn^2+^ is being displaced from the protein as a result of Cd^2+^ treatment (Figure 1D, red trace). There was no time-dependent increase in fluorescence for an identical experiment conducted in the absence of externally added Cd^2+^ (Figure 1D, blue trace), indicating that Fluozin-3 alone cannot strip Zn^2+^ off C1B. Collectively, these experiments show that Cd^2+^ successfully ejects Zn^2+^ from C1B and forms coordination bonds with cysteines.

### Cd^2+^ Binds to Both Cys_3_His Sites With the Formation of All-Cd and Cd/Zn Mixed C1B Species

**FIGURE 2:**
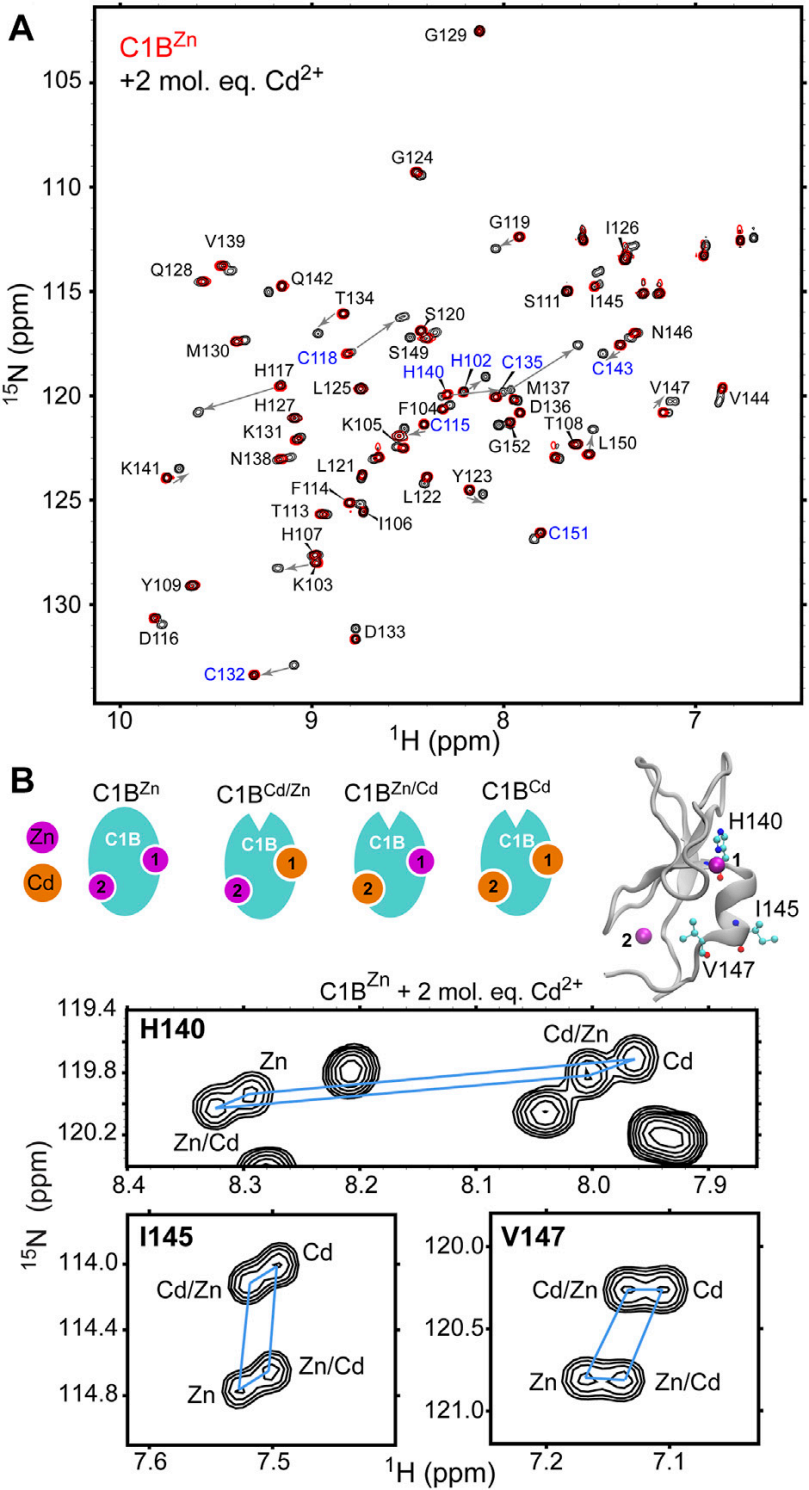
Cd^2+^ treatment results in the formation of fully Cd-bound and Zn/Cd mixed C1B species. **(A)** [^15^N- ^1^H] HSQC of 0.1 mM [U-^15^N] C1B^Zn^ by itself (red) and in the presence of 2 molar-equivalents of Cd^2+^ (black). Addition of Cd^2+^ results in an appearance of a new subset of cross-peaks. Arrows indicate the residue-specific changes in chemical shifts associated with Cd^2+^ binding to C1B. Zn^2+^-coordinating residues are highlighted in blue. **(B)** Expansions of the [^15^N-^1^H] HSQC spectra for three residues, His140, Ile145, and Val147 that show four distinct cross-peaks upon treatment of C1B^Zn^ with Cd^2+^. His140 is a Zn^2+^-coordinating residue; Ile145 and Val147 reside on the C-terminal α helix. The four Zn/Cd C1B species are shown in cartoon representation.

While the UV-vis data show that Cd^2+^ is displacing Zn^2+^ from C1B they do not contain any site-specific information. We used solution NMR spectroscopy to gain insight into how Cd^2+^ interacts with sites 1 and 2 of C1B (see Figure 1 for site definitions). The site-specific information was obtained by collecting 2D [^15^N, ^1^H] HSQC spectra of [U-^15^N] enriched C1B^Zn^ in the absence and presence of Cd^2+^. Each N-H group in C1B^Zn^ gives rise to a cross-peak in the 2D NMR spectra that we assigned in our previous work (Figure 2A, red spectrum) (Stewart et al., 2011). Upon addition of Cd^2+^, we observed an appearance of a new subset of well-dispersed C1B cross-peaks (Figure 2A, black spectrum). We were able to assign this subset to specific Cd/Zn C1B states based on their relative peak intensities and the chemical shifts of the refolded C1B^Cd^ (*vide infra*). From the spectral overlay, it is evident that the N-H resonances of many C1B residues, particularly those coordinating Zn1 and Zn2, experience large chemical shift perturbations upon C1B binding Cd^2+^.

In addition to native C1B^Zn^, there are three other possible Cd/Zn protein states: C1B^Cd^, C1B^Zn/Cd^, and C1B^Cd/Zn^ that can co-exist in solution. The N-H groups of three residues in C1B: His140, Ile145, and Val147 show four cross-peaks each (Figure 2B) and serve as direct evidence for the formation of the all-Cd and Zn/Cd mixed C1B species. Moreover, the distinct chemical shifts of the four cross-peaks enable the calculation of the relative affinities of Cd^2+^ to each metal ion coordination site, using the procedures described in the Materials and Methods section. The relative affinity data presented in Table 1 show that: (i) Cd^2+^ has a ∼2-fold and ∼1.6-fold higher affinities than Zn^2+^ for the C1B sites 1 and 2, respectively; and (ii) relative Cd^2+^ affinity for either site does not depend on the chemical identity of the metal ion, Cd^2+^ or Zn^2+^, that occupies the other site (i.e. for a given site the χ and μ values are essentially identical). We conclude that both thiol-rich coordination sites in C1B are reactive with respect to Cd^2+^ substituting for the native Zn^2+^ ion.

**TABLE 1:** Relative affinities of Cd^2+^ to the C1B Cys_3_His metal ion coordination sites.

*Residue*	*Cys*_*3*_ *His, site 1*		*Cys*_*3*_ *His, site 2*	
	*χ*[1]^a^	*μ*[1]^a^	*χ*[2]	*μ*[2]
H140	2.11	2.02	1.59	1.53
I145	1.74	1.81	1.51	1.57
V147	1.99	1.89	1.60	1.52
Mean^b^	1.94 ± 0.19	1.91 ± 0.11	1.57 ± 0.05	1.54 ± 0.03

a Relative affinities are calculated for the C1B states where one Cys_3_His site is already occupied by either Zn^2+^ (χ) or Cd^2+^ (μ).

b Error is reported as the standard deviation of the χ and μ values for the three residues.

**FIGURE 3:**
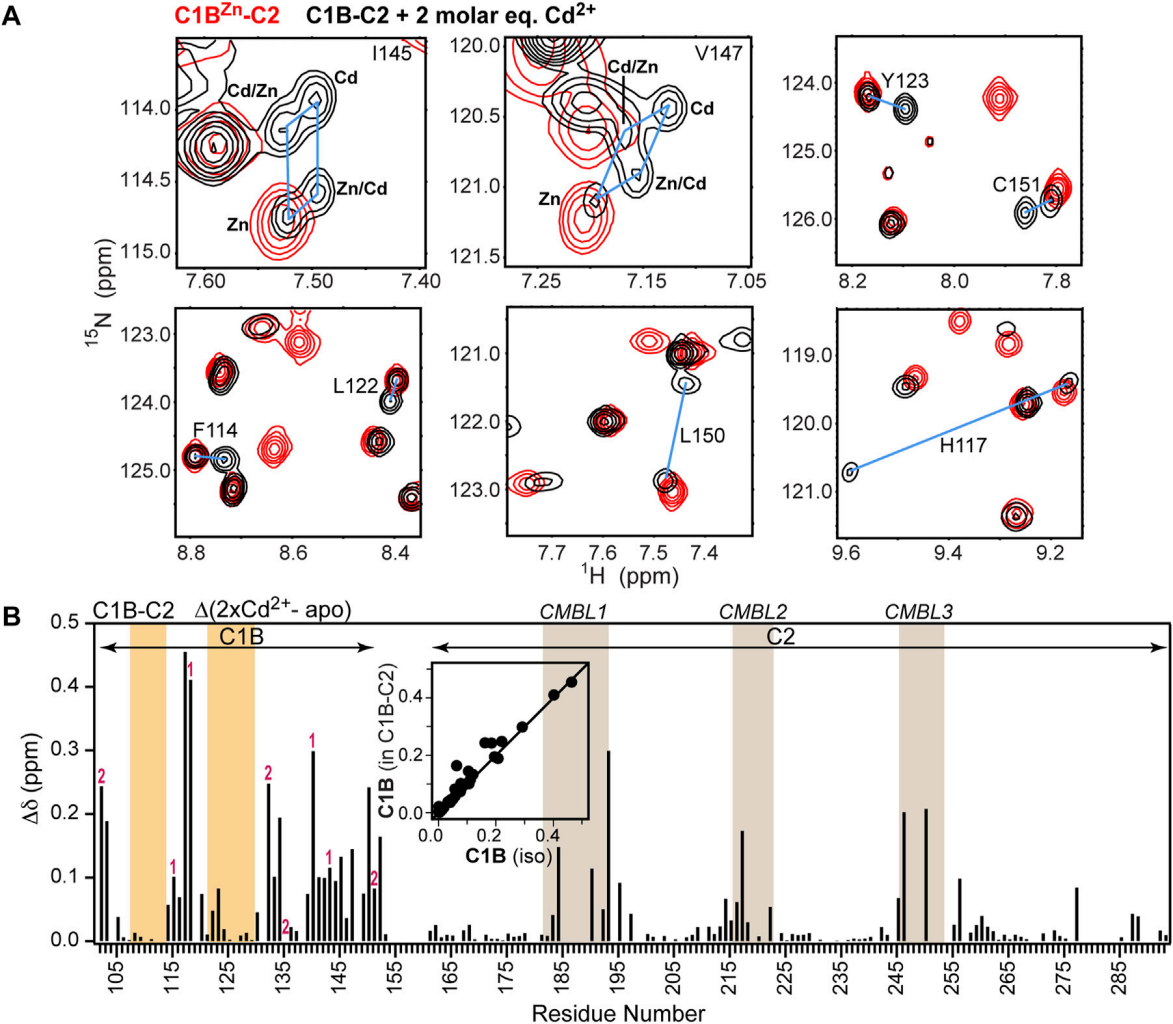
Cd^2+^ simultaneously populates thiol- and oxygen-rich sites in C1B-C2. **(A)** Overlays of the expansions of the [^15^N-^1^H] HSQC spectra of C1B^Zn^-C2 (red) and Cd^2+^-treated C1B-C2 (black). The N-H cross-peaks of Cd^2+^ and Zn^2+^ containing species are connected with blue lines. **(B)** Chemical shift perturbations (CSPs) for backbone N-H groups as a function of C1B-C2 primary structure. The CSP values were calculated between the C1B^Zn^-C2 and Cd^2+^-treated C1B-C2. Cys and His residues that coordinate Zn^2+^ in sites 1 and 2 are labeled accordingly. The C1B and C2 membrane-binding loops are highlighted in orange and tan, respectively. Inset: Correlation of C1B CSPs in the presence of 2 molar equivalents Cd ^2+^ in the isolated domain and in C1B-C2.

In the PKCα regulatory region, C1B is adjacent to the C2 domain. C2 is metal-ion free in the inactive state of the kinase, but binds Ca^2+^ that is released as a result of the signaling events preceding PKCα activation. The Ca^2+^ binding site is formed by the Ca^2+^- and membrane-binding loops or CMBLs (Figure 1A). To determine the effect of the C2 domain on the C1B-Cd^2+^ interactions, we compared the [^15^N,^1^H] HSQC spectra of C1B-C2 in the absence and presence of 2 molar equivalents of Cd^2+^. We observed the same signatures of Zn^2+^ replacement as in the isolated C1B domain, including the presence of four cross peaks for Ile145 and Val147 (Figure 3A). Overall, there is an excellent correlation between the chemical shift perturbations due to Cd^2+^ binding for isolated C1B and C1B in the context of its neighboring C2 (Figure 3B, inset). The full chemical shift perturbation (CSP) plot shows that not only C1B resonances are affected by interactions with Cd^2+^, but also the CMBLs of C2 (Figure 3B). We previously demonstrated that the isolated C2 domain can bind Cd^2+^ with high affinity (K_d_ < 1 μM) through the loop regions (Morales et al., 2013a). Collectively, these data indicate that Cd^2+^ binds simultaneously to both C1B and C2 domains and that the thiol-rich C1B Cys_3_His sites can effectively compete for Cd^2+^ with the C2 oxygen-rich sites.

### C1B Function Is Preserved Upon Zn^2+^ Replacement With Cd^2+^

**FIGURE 4:**
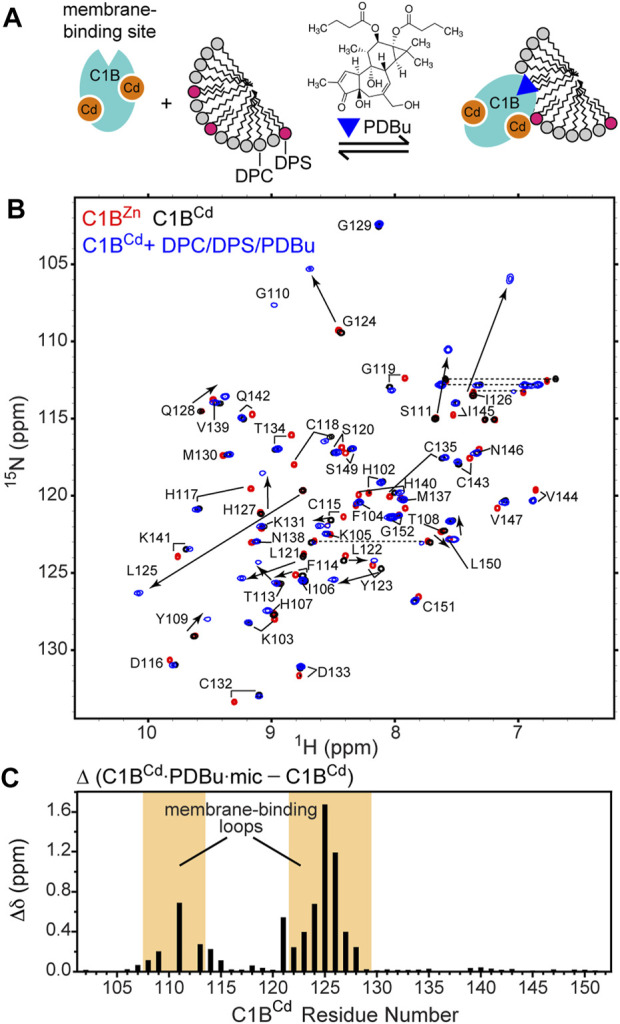
Cd^2+^ supports the PKC agonist-binding function of C1B. **(A)** Schematic representation of the experimental setup that involves C1B^Cd^, mixed micelles, and the PKC agonist PDBu. **(B)** [^15^N-^1^H] HSQC spectra of isolated native C1B^Zn^ (red), C1B^Cd^ (black), and C1B^Cd^ complexed to PDBu and mixed micelles (blue). **(C)** Chemical shift perturbations (CSPs) upon micelle/PDBu binding for the backbone N-H groups as a function of C1B primary structure. The membrane binding loops of C1B are highlighted in orange.

It is evident from the chemical shift dispersion in the 2D spectra that C1B remains folded upon incorporating Cd^2+^ (Figure 2 and Figure 3A). To test if C1B^Cd^ retains its function, we conducted NMR-detected binding experiments between C1B^Cd^ and a tumor-promoting agent, phorbol-12,13-dibutyrate (PDBu, Figure 4A). PDBu is an extremely potent exogenous agonist of PKC that binds specifically to C1 domains and drives their membrane insertion as part of the PKC activation sequence. These properties have made PDBu the most commonly used agonist (Katti and Igumenova, 2021) in the PKC field to assess the C1 domain functional competency. To generate C1B^Cd^ as the dominant species in solution, C1B^Zn^ was denatured and refolded in the presence of Cd^2+^. The 2D [^15^N,^1^H] HSQC spectrum of the refolded C1B^Cd^ showed distinct chemical shifts compared to those of C1B^Zn^ (Figure 4B), but superimposed exactly onto the spectrum of the Cd^2+^-bound species that were formed as a result of C1B^Zn^ treatment with Cd^2+^ (Figure 2A).

PDBu is an extremely hydrophobic ligand that requires a membrane-mimicking environment to form a soluble complex with C1 domains. To provide such an environment, we used the DPC/DPS mixed micelle system that supports the C1 ligand-binding function (Stewart et al., 2011; Stewart et al., 2014) and faithfully reproduces the outcomes of in-cell experiments. Upon addition of PDBu and mixed micelles to C1B^Cd^, we observed dramatic changes in the NMR spectrum (Figure 4B). Several residues, such as Ser111, Gly124, Leu125, and Ile126 experienced significant chemical shift perturbations upon the formation of the ternary C1B^Cd^-PDBu-micelle complex. The CSP plot comparing the complex with the apo state showed that the changes are localized to the C1B membrane-binding loop regions, which is responsible for capturing the ligand in the membrane environment (Figure 4C). This CSP pattern is essentially identical to that observed for the native C1B^Zn^ protein upon PDBu binding in micelles (Stewart et al., 2011). Because NMR chemical shifts are exquisitely sensitive to the electronic environment of the reporting nuclei, we conclude that C1B^Cd^ interacts with PDBu and partitions into micelles in a manner identical to that of the native C1B^Zn^.

### Kinetics of Cd^2+^ Binding Reports on the Inequivalency of the Cys_3_His Structural Sites

**FIGURE 5:**
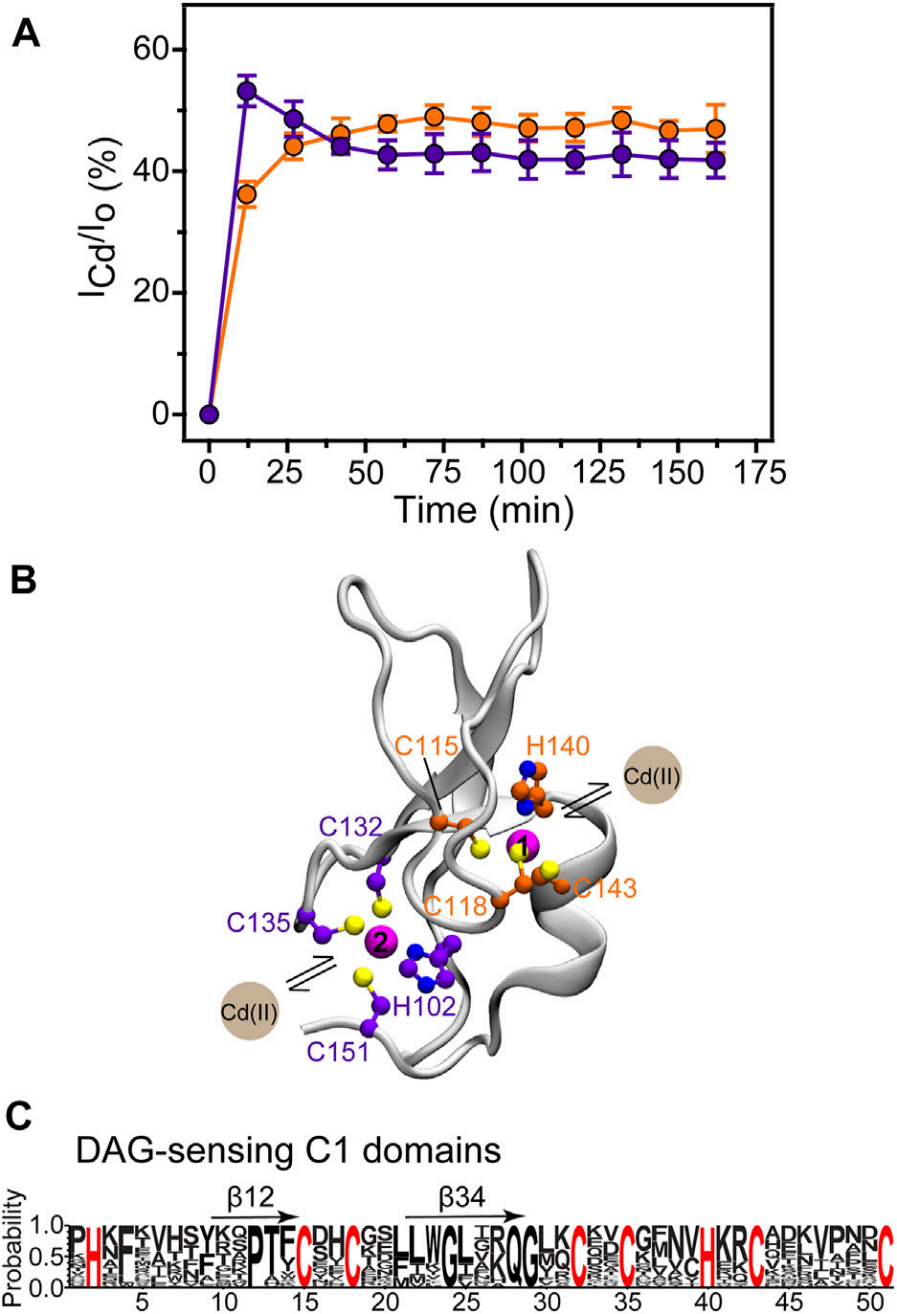
Site-resolved kinetics of Cd^2+^ binding to C1B. **(A)** The build-up of the Cd^2+^-bound C1B is plotted for the Cys_3_His sites 1 (orange) and 2 (purple). The error bars represent the standard deviations of the I_Cd_/I_0_ values within a given residue subset. **(B)** The residues that form sites 1 and 2 are highlighted on the ribbon diagram of C1B (2ELI). **(C)** WebLogo representations of the sequence alignment of 31 DAG-sensitive C1 domains found in DAG effector proteins (*R. norvegicus*). The sequence homology values are all between 52 and 92%. Cys_3_His motifs are strictly conserved. *β*12 and *β*34 denote the membrane-binding loops.

To investigate the site-specific kinetics of Zn^2+^ replacement with Cd^2+^, we used SOFAST HMQC experiments to monitor the build-up of the Cd^2+^-bound C1B species. The population in % was calculated as the ratio of the N-H cross-peak intensities of the Cd^2+^-bound C1B, I_Cd_, and the combined peak intensities I_0_ = I_Cd_ + I_Zn_. The data were plotted as the mean of the I_Cd_/I_0_ values for a subset of residues (listed in the Methods section) that report on Cd^2+^ binding to either site 1 or site 2. The kinetics data shown in Figure 5A revealed that sites 1 and 2 differ with respect to their kinetic behavior.

Site 2 is more reactive towards Cd^2+^, reaching the Cd^2+^-bound population of 53% within the first 15 min of the experiment. This exceeds the equilibrium value by ∼10%, and the site 1 population by 17%. As shown on the 3D structure of the C1B domain in Figure 5B, Zn^2+^ at site 2 brings the termini of C1B together by coordinating His102 at the N-terminus and Cys151 at the C-terminus. This part of the protein has a relatively high degree of solvent exposure and is therefore readily accessible to Cd^2+^. Another distinct feature of site 2 is the presence of a reactive Cys residue, Cys151, which serves as the entry point for the reactive oxygen species that activate PKCα in a process involving Zn^2+^ release. The structural dynamics of site 2, associated with the loss of Cys151 coordination bond with Zn^2+^ (Stewart and Igumenova, 2012), is likely to be another factor that makes site 2 susceptible to Cd^2+^ interactions. Under the conditions of our experiment, the system reached equilibrium within 1 hour. At equilibrium, the Cd^2+^ population of site 1 is higher than that of site 2, fully consistent with the pattern of relative Cd^2+^ affinities (Table 1). Together, the data of Figure 1D, 5(A), and Table 1 show that Cd^2+^ binding accompanied by Zn^2+^ ejection is a slow process, and that sites 1 and 2 are non-equivalent kinetically and thermodynamically.

## Discussion

Due to the similarities in charge and ligand preferences, xenobiotic Cd^2+^ ions target proteins that rely on Ca^2+^ and Zn^2+^ for their function (Choong et al., 2014; Petering, 2017; Duan et al., 2018; Ben Mimouna et al., 2019). Cd^2+^ has high affinity for thiol groups (Krizek et al., 1993) and, just like Zn^2+^, prefers tetrahedral geometry when coordinated by sulfur and nitrogen ligands. Cd^2+^ interactions with thiol groups in proteins were proposed to drive aggregation of nascent proteins through inhibition of folding *in vitro* (Sharma et al., 2008) and *in vivo* (Jacobson et al., 2017), whereas treatment with Zn^2+^ was shown to have a protective effect. Cd^2+^ can also target protein oxygen-rich sites and engage in either specific interactions in lieu of Ca^2+^ (Morales et al., 2013a; Katti et al., 2017) or opportunistic interactions that result in the formation of well-defined protein aggregates (Cole et al., 2019).

Here, we used Cd^2+^ to probe the reactivity of the structural Zn^2+^ sites in the regulatory membrane-binding region of the Ca^2+^-activated Protein Kinase Cα. Previous work on Zn^2+^ replacement by Cd^2+^ at protein structural sites suggests that generally this process can have varying consequences for the protein structure and function (Pan et al., 1990; Huang et al., 2004; Michalek et al., 2012). While in some cases Cd^2+^ was demonstrated to support the protein fold and function (Malgieri et al., 2014), global structural rearrangements due to Cd^2+^ replacing Zn^2+^ and loss of or change in function were also reported (Huang et al., 2004; Malgieri et al., 2011; Michalek et al., 2012). The use of Cd^2+^ in folding the C1 peptides derived from PKCα, β, and γ isoforms revealed isoform-specific differences in the functional behavior (Irie et al., 1998) and highlighted the need to investigate the Cd^2+^ response in the context of the fully folded PKC regulatory region that harbors all potential metal-ion binding sites. To that end, we used the C1B-C2 membrane-binding regulatory region to evaluate the site-specific response and reactivity of the structural thiol-rich Zn^2+^ sites towards Cd^2+^ substitution in the context of the neighboring Ca^2+^-sensing C2 domain. Despite the presence of competing oxygen-rich C2 metal ion binding sites, Cd^2+^ was able to partially eject Zn^2+^ from C1B-C2 (Figure 1D) with the formation of the all-Cd and Zn/Cd mixed metal ion protein species (Figure 3A). The solution NMR approach was critical here, as it enabled us to follow the Cd^2+^-binding process in the site-specific manner, starting with the fully folded domains and a native Zn^2+^ ion populating the C1B structural sites.

By specifically focusing on the isolated C1B domain, we were able to identify the spectroscopic signatures of Zn^2+^ replacement with Cd^2+^ (Figure 2) and use them to obtain thermodynamic and kinetic properties of the two Cys_3_His sites. The Cd^2+^ replacement occurs spontaneously, due to the ∼1.6- (site 2) and ∼2-fold (site 1) higher affinity to Cd^2+^ relative to Zn^2+^ (Table 1). The relative affinities can be explained by Cd^2+^ being a softer Lewis acid (larger ionic radius and polarizability) than Zn^2+^ and therefore forming stronger interactions with thiolate ligands (Puljung and Zagotta, 2011). This property confers thermodynamic advantages onto Cd^2+^ interactions with protein sites that are thiol-rich, such as Cys_3_His and Cys4 (Kluska et al., 2018). With respect to the Cd^2+^/Zn^2+^ replacement kinetics (Figure 1D and Figure 5), the reaction is slow to reach full equilibrium, likely due to the small Zn^2+^ k_off_ rate constants that are typical for the high-affinity structural sites. Despite the coordination spheres being identical, site 2 is more reactive with respect to Cd^2+^ binding. This is evidenced by the sharp increase in the respective population of Cd^2+^-bound C1B species that get equilibrated within an hour to form all four possible metal-ion bound states (Figure 5A). We attribute the reactivity of site 2 to Cd^2+^ to its higher solvent exposure and the presence of the reactive Cys residue, Cys 151, in the coordination sphere. We previously demonstrated that in addition to being susceptible to oxidation and alkylation, Cys151 undergoes a dynamic process that slightly opens up site 2 of the C1B structure (Stewart and Igumenova, 2012). Given that this cysteine residue is proposed to be the PKC entry point of reactive oxygen species, we speculate that Cd^2+^ could have a protective effect by forming a stronger bond with the Cys151 residue. The diacylglycerol-sensitive C1 domains share significant sequence homology (Figure 5C), and the two Zn^2+^-coordinating Cys_3_His motifs are strictly conserved. This strongly suggests that our findings on the reactivity of the Cys_3_His sites in C1B from PKCα are broadly applicable to the other C1 domains. It remains to be established if the other C1s show a similar pattern of relative site reactivity, with site 2 being more reactive than site 1.

Our results for the regulatory region of PKC suggest a possible explanation of how Cd^2+^ can modulate PKC activity. Cd^2+^ spontaneously incorporates itself into the C1B structural sites without compromising the fold and PDBu-binding (Figure 4). It is therefore likely that Cd^2+^-substituted C1 domains will retain at least part of their diacylglycerol-binding function. The membrane-binding function of Ca^2+^-responsive C2 domains, however, is inhibited by Cd^2+^—despite its relatively high-affinity to the oxygen-rich sites of the C2 membrane-binding loops (Morales et al., 2013a; Katti et al., 2017). Since the membrane association of both domains is necessary for PKC activation, the inhibitory effect of C2 might be predominant at high Cd^2+^ concentrations. These findings may also have implications for the mechanisms of Cd^2+^ toxicity in the cell, where the identity and occupancy of target protein sites will depend on the concentration of bioavailable Cd^2+^.

## Materials and Methods

### Buffers and Metal Ion Stock Solutions

The Cd^2+^ stock solutions were prepared by dissolving Cd(NO_3_)_2_·4H_2_O (>99% purity, Sigma-Aldrich) in the appropriate buffer. Unless indicated otherwise, the experiments were conducted in the “MES buffer” comprising 10 mM 2-(N-morpholino)ethanesulfonic acid (MES) at pH 6.0 in HPLC-grade water (Avantor), 150 mM KCl, and 1 mM tris(2-carboxyethyl) phosphine (TCEP). The buffers were passed through the Chelex^®^ 100 (Sigma-Aldrich) column to remove residual divalent metal ions.

### Protein Expression

The DNA sequences encoding C1B-C2 (residues 100–293), isolated C1B (residues 100–152) or C2 (residues 155–293) of PKCα (*M. musculus* for C1B-C2 and C1B; *R. Norvegicus* for C2) were amplified by PCR using the cDNA clone of PKCα (Open Biosystems) as a template and cloned into the pET-SUMO vector (Invitrogen). Isolated C1B, C2, and C1B-C2 were expressed and purified as described previously (Morales et al., 2011; Stewart et al., 2011; Cole et al., 2019). [U-^15^N, 75%-^2^H]-enriched C1B-C2 and [U-^15^N]- or [U-^13^C, ^15^N]-enriched C1B were used for the NMR experiments.

### UV-Vis Spectroscopy

UV-vis spectra were collected on a Beckman DU 640 spectrophotometer. 25 μM protein (C1B-C2, C2, or C1B) solution or MES buffer (for metal ion-only reference experiments) were placed in the sample cuvette; the reference cuvette always contained metal ion-free MES buffer. Cd^2+^ was added stepwise from the corresponding stock solutions to the sample cuvette. The samples were incubated for 1 h prior to the start of the measurements. The post-acquisition processing included the subtraction of the free Cd^2+^ spectra from the spectra of protein-containing samples. To eliminate contribution of protein-only absorption bands, the difference spectra were generated by subtracting the spectrum of the apo protein from the spectra of the metal-ion-containing protein. All spectra were corrected for dilution prior to subtraction.

### C1B Refolding

[U-^15^N]-enriched C1B was dissolved in 6 M guanidine hydrochloride (Acros Organics) and the “refolding buffer” comprising 20 mM MES at pH 6.0 and 1 mM TCEP. The final protein concentration was between 15 and 35 μM during the denaturation step. The refolding was conducted in three dialysis steps, all of them carried out in the refolding buffer: (1) against 8 M urea at room temperature, for 8 h; (2) against 1.5 M urea and 100 μM Cd(II) nitrate at 4°C, overnight; and (3) against urea-free buffer at 4°C, for 3 days to ensure complete removal of urea. The refolded protein was concentrated in a Vivaspin^®^ spin concentrator with a 3 kDa cut-off and subsequently exchanged into an “NMR buffer” (10 mM MES at pH 6.0, 150 mM KCl, 1 mM TCEP, 0.02% NaN_3_, and 8% (v/v) D_2_O using a Midi-Trap G25 desalting column (GE Healthcare).

### NMR Spectroscopy

All proteins were concentrated and buffer exchanged using 10 kDa (C1B-C2), 3 kDa (C1B) and 5 kDa (C2) cut-off Vivaspin® 15R concentrators into an NMR buffer. The experiments were carried out on Avance III HD NMR spectrometers (Bruker Biospin), operating at the ^1^H Larmor frequencies of 800 MHz (18.8 Tesla) and 600 MHz (14.1 Tesla) equipped with cryogenically cooled probes, and 500 MHz (11.7 Tesla) equipped with a room temperature probe. The temperature was calibrated using deuterated (D4, 98%) methanol for cryogenically cooled probes and protonated methanol for the room temperature probe. Spectra were processed using NMRPipe (Delaglio et al., 1995). The cross-peak intensities were obtained using Sparky (Si et al., 2015). Sequence-specific assignments of the ^1^H_N_ and ^15^N resonances for apo C1B-C2 were obtained using ^2^H-decoupled 3D HN(CA)CB, HNCA(CB), HN(COCA)CB, and HN(CO)CA (Yamazaki et al., 1994) experiments on a [U-^13^C,^15^N; 55%-^2^H] C1B-C2 sample. Resonance assignments for Cd^2+^-substituted C1B (C1B^Cd^) were transferred from those for the native Zn^2+^-containing protein (C1B^Zn^) and subsequently verified using 3D CBCA(CO)NH and HNCACB (Muhandiram and Kay, 1994) spectra collected at 14.1 Tesla. Resonance assignments for Cd^2+^-bound C1B-C2 were transferred from those for the isolated C1B^Cd^ and the Cd^2+^-complexed C2 (Morales et al., 2013b) domains. Chemical shift perturbations Δ were calculated between Cd^2+^-free and Cd^2+^-containing C1B-C2 as well as micelle/PDBu bound C1B^Cd^ and apo C1B^Cd^ according to the following equation:

$$\begin{array}{l} \Delta =\sqrt{\Delta \delta_{H}^{2}+{\left(0.152\Delta \delta_{N}\right)}^{2}} \end{array}$$ (1)

where Δδ_H_ and Δδ_N_ are residue-specific ^1^H_N_ and ^15^N chemical shift differences. For the NMR-detected binding experiments, the C1B ligand, phorbol-12,13-dibutyrate (PDBu, Sigma-Aldrich) was dissolved in [^2^H_6_] DMSO (Cambridge Isotopes) and added to the sample containing 94 μM of [U-^15^N] enriched C1B^Cd^ in the presence of 10 mM mixed micelles. Mixed micelles comprising [^2^H_38_] dodecylphosphocholine, (DPC, Cambridge Isotopes) and 2-dihexanoyl-sn-glycero-3-[phospho-l-serine] (DPS, Avanti Polar Lipids) at a molar ratio of seven to three were prepared as previously described (Stewart et al., 2011). The final concentration of PDBu in the NMR sample was 100 μM.

### Determination of Relative Cd^2+^ and Zn^2+^ Affinities to C1B

The four possible Zn/Cd metallated protein states are identified using the following nomenclature: C1B^Zn^ (native C1B with Zn^2+^ at both structural sites), C1B^Cd^ (Cd^2+^ at both structural sites), C1B^Zn/Cd^ (Zn^2+^ at site 1 and Cd^2+^ at site 2), and C1B^Cd/Zn^ (Cd^2+^ at site 1 and Zn^2+^ at site 2). The fractional populations of those protein species can be defined as:

$$\begin{array}{l} f_{Zn}=\frac{I_{Zn}}{\Sigma },f_{Zn/Cd}=\frac{I_{Zn/Cd}}{\Sigma },f_{Cd/Zn}=\frac{I_{Cd/Zn}}{\Sigma },f_{Cd}=\frac{I_{Cd}}{\Sigma }\\ \Sigma =I_{Zn}+I_{Zn/Cd}+I_{Cd/Zn}+I_{Cd} \end{array}$$ (2)

where *I* is the intensity of the corresponding N-H cross peaks in the ^15^N-^1^H HSQC spectra for H140, I145, and V147. The concentrations of free Cd^2+^ ([Cd^2+^]) and Zn^2+^ ([Zn^2+^]) can be calculated from the following mass balance equations:

$$\frac{{\left[Cd^{2+}\right]}_{0}}{P_{0}}=\frac{\left[Cd^{2+}\right]}{P_{0}}+2f_{Cd}+f_{Zn/Cd}+f_{Cd/Zn}$$ (3)

$$2=\frac{\left[Zn^{2+}\right]}{P_{0}}+2f_{Zn}+f_{Zn/Cd}+f_{Cd/Zn}$$ (4)

where P_0_, [Cd^2+^]_0_, and [Zn^2+^]_0_ = 2×P_0_ are the total concentrations of protein, Cd^2+^, and Zn^2+^, respectively. It is convenient to define the affinities of metal ions to C1B in terms of individual sites. For the single metal-ion bound species, we use the M [n] notation, where M = Zn or Cd, and n = 1 or 2. For example, C1B^Zn[2]^ defines C1B with site 2 populated by Zn^2+^ and a vacant site 1, and K_a_^Zn[1]^ defines the association constant for the binding of Zn^2+^ to site 1 when site 2 is already populated by Zn^2+^. The following equilibria describe the binding processes and the associated K_a_ values:

$$\begin{array}{l} C1B^{Zn\left[2\right]}+Zn^{2+}\overset{K_{a}^{Zn\left[1\right]}}{⇌}C1B^{Zn}\;\;\;\;\;\;\;K_{a}^{Zn\left[1\right]}=\frac{\left[C1B^{Zn}\right]}{\left[C1B^{Zn\left[2\right]}][Zn^{2+}\right]} \end{array}$$ (5)

$$C1B^{Zn[1]}+Zn^{2+}\overset{K_{a}^{Zn\left[2\right]}}{⇌}C1B^{Zn}\;\;\;\;K_{a}^{Zn\left[2\right]}=\frac{\left[C1B^{Zn}\right]}{\left[C1B^{Zn[1]}][Zn^{2+}\right]}$$ (6)

$$C1B^{Zn[2]}+Cd^{2+}\overset{K_{a}^{Cd\left[1\right]}}{⇌}C1B^{Cd/Zn}\;\;\;\;K_{a}^{Cd\left[1\right]}=\frac{\left[C1B^{Cd/Zn}\right]}{\left[C1B^{Zn[2]}][Cd^{2+}\right]}\;$$ (7)

$$C1B^{Zn[1]}+Cd^{2+}\overset{K_{a}^{Cd\left[2\right]}}{⇌}C1B^{Zn/Cd}\;\;\;\;K_{a}^{Cd\left[2\right]}=\frac{\left[C1B^{Zn/Cd}\right]}{\left[C1B^{Zn[1]}][Cd^{2+}\right]}$$ (8)

The relative affinity of Cd^2+^ and Zn^2+^ to sites 1 and 2 can then be defined as the ratio of the association constants:

$$\chi \left[1\right]=\frac{K_{a}^{Cd\left[1\right]}}{K_{a}^{Zn\left[1\right]}}=\frac{\left[Zn^{2+}\right]f_{Cd/Zn}}{\left[Cd^{2+}\right]f_{Zn}}$$ (9)

$$\chi \left[2\right]=\frac{K_{a}^{Cd\left[2\right]}}{K_{a}^{Zn\left[2\right]}}=\frac{\left[Zn^{2+}\right]f_{Zn/Cd}}{\left[Cd^{2+}\right]f_{Zn}}$$ (10)

The χ[n] (n = 1 or 2) values report on the relative affinities of Cd^2+^ and Zn^2+^ to a given site C1B site when Zn^2+^ populates the other. A similar set of equilibria can be constructed to obtain the relative Cd^2+^ and Zn^2+^ affinities when Cd^2+^ populates the other site:

$$\mu \left[1\right]=\frac{\left[Zn^{2+}\right]f_{Cd}}{\left[Cd^{2+}\right]f_{Zn/Cd}}$$ (11)

$$\mu \left[2\right]=\frac{\left[Zn^{2+}\right]f_{Cd}}{\left[Cd^{2+}\right]f_{Cd/Zn}}$$ (12)

χ[n] and μ[n] for sites 1 and 2 (Table 1) were calculated using the NMR cross-peak intensities and the total concentrations of Cd^2+^, C1B, and Zn^2+^ in the system (see Eqs. 1–3). The NMR cross-peaks intensities were determined using the [^15^N-^1^H] HSQC spectrum of 0.1 mM [U-^15^N] C1B^Zn^, equilibrated overnight in the presence of 0.1 mM Cd^2+^.

### Site-specific Kinetics of Cd^2+^ Binding to C1B

To monitor the kinetics of Cd^2+^ binding to C1B, 2-fold molar excess of Cd^2+^ was added to 200 μM [U-^13^C,^15^N; ∼75%-^2^H] C1B^Zn^ in 10 mM HEPES buffer at pH 7.2, 75 mM KCl, and 1 mM TCEP. The process of Zn^2+^ replacement with Cd^2+^ was monitored using SOFAST-HMQC NMR experiments that were conducted on a 500 MHz instrument (11.7 Tesla) equipped with a room temperature probe. The first time point started 12 min post Cd^2+^ addition, and each SOFAST HMQC experiment took 15 min. Because the inter-conversion between Zn^2+^- and Cd^2+^-complexed states is in the slow exchange regime, at any time point their fractional population can be determined from the intensities of the corresponding amide cross-peaks in the SOFAST-HMQC spectra. We used the N-H resonances of His102, Cys132, Thr134, Cys135, and Leu150 as the reporters of Cd^2+^ binding to site 1; and Phe114, Cys115, His117, Cys118, Gly119, Ser120, Tyr123, Lys141, and Cys143 as the reporters of Cd^2+^ binding to site 2.

## Data Availability

The original contributions presented in the study are included in the article/Supplementary Material, further inquiries can be directed to the corresponding author.
